# Supplementary material for: Changes in Sphingolipid Profile of Benzo[a]pyrene-Transformed Human Bronchial Epithelial Cells Are Reflected in the Altered Composition of Sphingolipids in Their Exosomes
Source: Int J Mol Sci. 2021 Aug 25;22(17):9195. doi: 10.3390/ijms22179195 (PMC8431443; doi:10.3390/ijms22179195)
Supplement: Supplementary file 1 [file ijms-22-09195-s001.zip › ijms-1335032-supplementary.pdf]

Supplementary Information

Machala et al.: Transformation of human bronchial epithelial cells induced by benzo[a]pyrene is reflected in altered lipidomic profile of exosomes

Supplementary Table S1.
Supplementary Table S2.
Supplementary Table S3.
Supplementary Figure S1.

Supplementary Table S1. List of internal standards for SL and GSL analyses.

Avanti Polar Lipids Inc., lot number LM6005, Ceramide/Sphingoid Internal Standard Mix II

Lipid species	Abbreviation	Molecular ion [m/z]	MW
C12 sphingomyelin	SM 12-0	647.3	646.3
C12 ceramide	Cer 12-0	482.4	481.4
C12 Glucosyl(β) - ceramide	GlcCer 12-0	644.6	643.6
C12 lactosyl(β) - ceramide	LacCer 12-0	806.6	805.6
C12 ceramide-1-phosphate	CerP 12-0	562.4	561.4
C17 sphingosine	So 17-1	286.3	285.3
C17 sphinganine (dihydro-sphingosine)	Sa 17-0	288.3	287.3
C17 sphingosine-1-phosphate	So 17-1-P	366.3	365.3
C17 sphinganine-1-phosphate (dihydro-sphingosine-1-phosphate	So 17-1-P	368.2	367.2

Avanti Polar Lipids Inc., lot numbers 860573P and 860073W

C12 Mono-Sulfo galactosyl(β) Ceramide (d18:1/12:0)	Sulf 12/0	725.1	741.0
C18:0 GM3-d5 (synthetic)	GM3 18/0 d5	594.3	1203.5

Supplementary Table S2. List of external standards for eicosanoid analysis (Cayman Chemical Company, Ann Arbor, MI, USA).

Lipid species	Abbreviation	Molecular ion [m/z]	MW
6 – keto prostaglandin $F_{1\alpha}$	6-keto-$PGF_{1\alpha}$	370.2	370.5
prostaglandin D2	PGD_2	352.2	352.5
prostaglandin E_2	PGE_2	352.2	352.5
prostaglandin A_2	PGA_2	334.2	334.5
prostaglandin J_2	PGJ_2	334.2	334.5
prostaglandin $F_{2\alpha}$	$PGF_{2\alpha}$	354.2	354.5
prostaglandin $F_{2\beta}$	$PGF_{2\beta}$	354.2	354.5
8-iso prostaglandin $F_{2\beta}$	8-Iso- $PGF_{2\beta}$	354.2	354.5
15-keto prostaglandin E_2	15-keto- PGE_2	350.2	350.5
13,14-dihydro-15- keto prostaglandin E2	13,14-DH-15-keto-PGE_2	352.2	352.5
13,14-dihydro-15- keto prostaglandin D2	13,14-DH-15-keto-PGD_2	352.2	352.5
Lipoxin A4	LXA_4	352.2	352.5
20- hydroxyeicosatetraenoic acid	20-HETE	320.2	320.5
15- hydroxyeicosatetraenoic acid	15-HETE	320.2	320.5
8- hydroxyeicosatetraenoic acid	8-HETE	320.2	320.5
12- hydroxyeicosatetraenoic acid	12-HETE	320.2	320.5
13-hydroxyoctadecadienoic acid	13-HODE	296.2	296.5
9-hydroxyoctadecadienoic acid	9-HODE	296.2	296.5
Arachidonic acid	AA	304.2	304.5

Supplementary Table S3. Primers and probes for RT-qPCR.

Gene	TaqMan assay ID
UGCG	Hs00916612_m1
UTG8	Hs00409958_m1
GALC	Hs00164660_m1
GBA	Hs00986836_g1
GBA2	Hs01107320_g1
B4GALT5	Hs00941041_m1
B4GALT6	Hs00999574_m1
GLA	Hs00609238_m1
GAL3ST1	Hs00191582_m1
CERT	Hs01062552_m1
FAPP2	Hs01696164_s1
ST3GAL5	Hs01105377_m1
NEU3	Hs00198406_m1
B4GALNT1	Hs01110791_g1
B3GALT4	Hs00534104_s1
HEXA	Hs00942655_m1
HEXB	Hs01077594_m1
ST8SIA1	Hs01124292_m1
ST8SIA4	Hs00379924_m1
ST8SIA5	Hs00203298_m1
ST8SIA6	Hs02341869_g1
A4GALT	Hs05058505_s1
B3GALNT1	Hs00364202_sl
PTGDS	Hs00168748_m1
PTGES	Hs00610420_m1
PTGES2	Hs00228159_m1

Gene	Nucleotide acc.no.	Primers		UPL probe no.
		forward	reverse	
B3GNT5	NM_032047.4	tcaaaactctgtttgccttagga	cccaagccagttttctttgt	7
PTGS2	NM_000963.2	cttcacgcatcagtttttcaag	tcaccgtaaatatgatttaagtccac	23
GLB1	Assay ID 115378 GLB1 human; kat. number 05532957001 (Roche)			-

Supplementary Figure S1. Differences in SL (A) and GSL (B) species in BaP-transformed and parental HBEC-12KT cells and their exosomes. The levels of SL/GSL were measured by LC/MS-MS and are expressed relative to parental HBEC-12KT as means ± standard deviation obtained from three independent experiments performed in triplicates for cells and five experiments performed in monoplicates for exosomes. The asterisks denote a significant difference between the effects *p<0.05, **p< 0.01.

A)

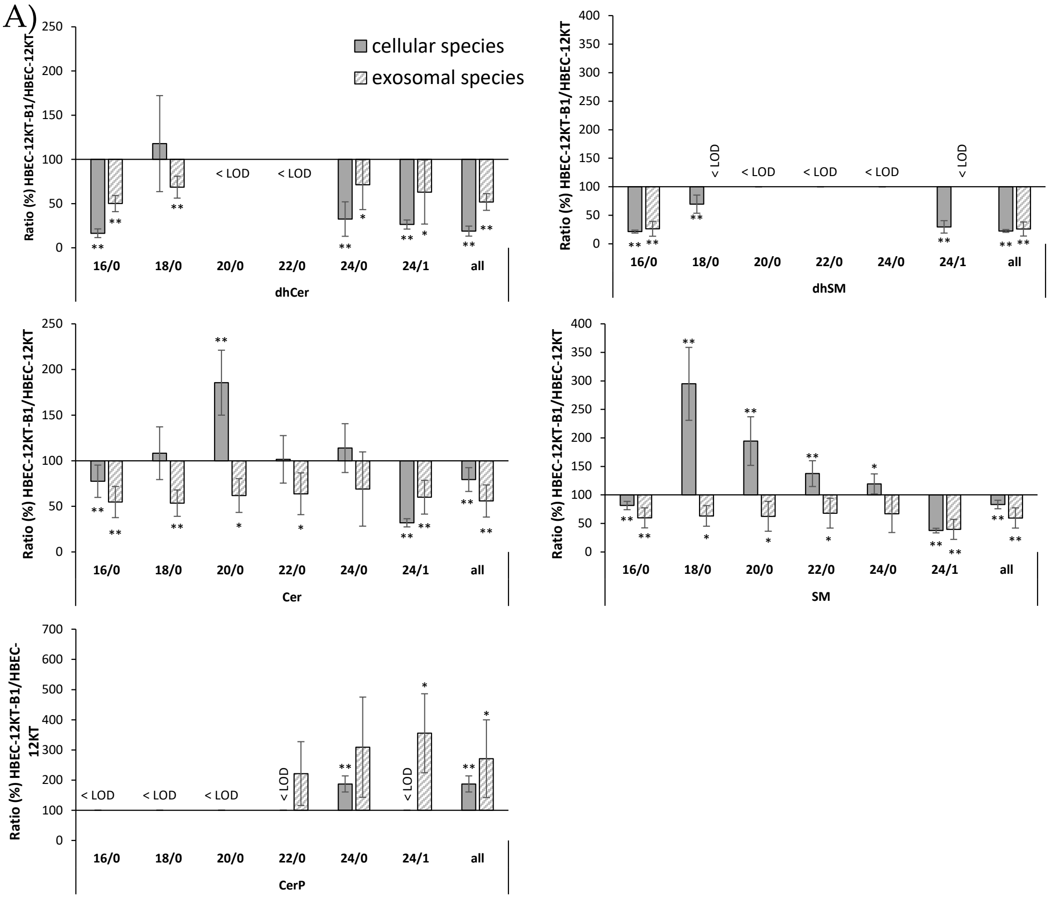

B)

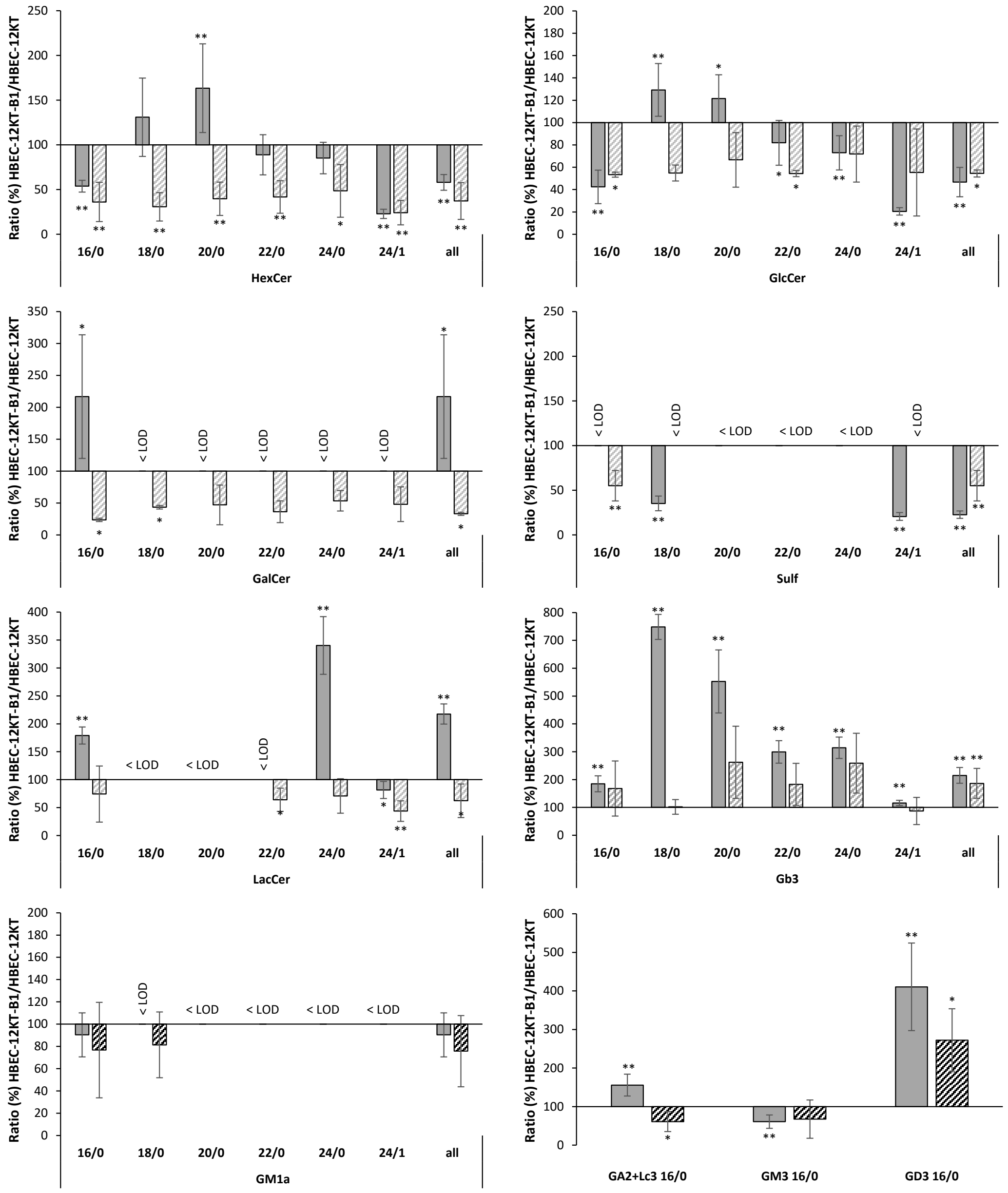
Ratio (%) HBEC-12KT-B1/HBEC-12KT
16/0
18/0
20/0
22/0
24/0
24/1
all
HexCer
GlcCer
GalCer
Sulf
LacCer
Gb3
GM1a
< LOD
GA2+Lc3 16/0
GM3 16/0
GD3 16/0
